# Dynamics and Regulations of BimEL Ser65 and Thr112 Phosphorylation in Porcine Granulosa Cells during Follicular Atresia

**DOI:** 10.3390/cells9020402

**Published:** 2020-02-10

**Authors:** Feng Yang, Yanhong Chen, Qiang Liu, Shizhen Dai, Shenming Zeng

**Affiliations:** National Engineering Laboratory for Animal Breeding, Key Laboratory of Animal Genetics and Breeding of the Ministry of Agriculture, College of Animal Science and Technology, China Agricultural University, Beijing 100083, China; yangcfeng@cau.edu.cn (F.Y.); chenyanhong2017@outlook.com (Y.C.); liuqiangtaian2008@163.com (Q.L.); daishizhen34@163.com (S.D.)

**Keywords:** BimEL phosphorylation, porcine, follicle atresia, granulosa cell, apoptosis

## Abstract

BimEL protein is involved in follicular atresia by regulating granulosa cell apoptosis, but the dynamic changes of BimEL phosphorylation during follicular atresia are poorly understood. The aim of this study was to explore the changes of key BimEL phosphorylation sites and their upstream regulatory pathways. First, the levels of BimEL-Ser65 and BimEL-Thr112 phosphorylation (p-BimEL-S65, p-BimEL-T112) in granulosa cells (GC) from healthy (H), slightly-atretic (SA), and atretic (A) follicles and in cultured GC after different treatments were detected by Western blotting. Next, the effects of the corresponding site mutations of *BIM* on apoptosis of GC were investigated. Finally, the pathways of two phosphorylation sites were investigated by kinase inhibitors. The results revealed that p-BimEL-S65 levels were higher in GC from H than SA and A, whereas p-BimEL-T112 was reversed. The prosurvival factors like FSH and IGF-1 upregulated the level of p-BimEL-S65, while the proapoptotic factor, heat stress, increased the level of p-BimEL-T112 in cultured GC. Compared with the overexpression of wild BimEL, the apoptotic rate of the GC overexpressed BimEL-S65A (replace Ser65 with Ala) mutant was significantly higher, but the apoptotic rate of the cells overexpressing BimEL-T112A did not differ. In addition, inhibition of the ERK1/2 or JNK pathway by specific inhibitors reduced the levels of p-BimEL-S65 and p-BimEL-T112. In conclusion, the levels of p-BimEL-S65 and p-BimEL-T112 were reversed during follicular atresia. Prosurvival factors promote p-BimEL-S65 levels via ERK1/2 to inhibit GC apoptosis, whereas proapoptotic factor upregulates the level of p-BimEL-T112 via JNK to induce GC apoptosis.

## 1. Introduction

Although follicle development is an ongoing process, atresia prevents follicles from ovulating to affect reproductive activity. In mammals, more than 99% of developing follicles undergo atresia [1,2,3]. It is widely accepted that ovarian follicular atresia is primarily caused by granulosa cell apoptosis [2], which is regulated by internal and external factors [4]. Two general pathways have been reported to be involved in apoptosis: survival factors withdrawal and bindings of specific dead ligands, such as tumor necrosis factor-alpha or Fas ligand, to their membrane receptors [5]. In mammalian ovarian follicles, granulosa cell apoptosis is triggered when endocrine and/or intrafollicular survival factors are inadequate, particularly gonadotropins and some growth factors [6,7]. According to a recent transcriptome analysis on porcine follicular atresia, *DKK3*, *GADD45A*, *CAMTA2*, *CCDC80*, *DAPK2*, *ECSIT*, *MSMB*, *NUPR1*, *RUNX2*, *SAMD4A*, and *ZNF628* having a fold-change higher than five between healthy and atretic follicle granulosa cells could likely serve as markers of pig follicular atresia [7]. The let-7 miRNA family can also be related to granulosa cell programmed death, and let-7a/b/c/i may target TP53, CASP3, and FAS to prevent apoptosis, while let-7g may induce apoptosis by binding to CCND2 or Bcl-XL [8].

The Bcl-2 protein family plays irreplaceable roles during apoptosis, and one of the most important proteins is the BH3-only protein, Bim. Bim binds with high affinity to antiapoptotic Bcl-2 family members and regulates apoptotic signaling through Bax and Bak [9]. The gene encoding the Bim protein can be translated into a variety of homologs, including BimEL, BimL, and BimS, among which BimEL is the most abundant in cells [10]. BimEL has at least eight phosphorylation sites, which endow its different functions [10,11]. For example, the phosphorylation of BimEL at Ser65 is required for rapid dissociation of BimEL/Mcl-1 and BimEL/Bcl-xL complexes [12], which may play a vital role in BimEL degradation via the proteasome pathway to promote cell survival [13,14,15]. The stress kinase JNK can phosphorylate BimL at Thr56 and BimEL at Ser100, Thr112, and Ser114, which reduces the binding of BimEL to DLC1 (dynein light chain 1), leading to cell apoptosis [16,17,18,19]. Our recent results demonstrated that heat stress promotes BimEL phosphorylation through the JNK pathway and decreases the level of aromatase in porcine granulosa cells to damage follicular development [20]. Our prevous work also showed that IGF-1, insulin, and melatonin could phosphorylate and downregulate BimEL protein level, which can inhibit apoptosis of porcine granulosa cell [13,21,22].

During the process of follicular atresia, the level of BimEL protein in porcine granulosa cells is elevated [23], but the BimEL phosphorylation profile in granulosa cells is unknown during this process. In this experiment, the dynamics and regulations of BimEL, Ser65, and Thr112 phosphorylation during follicular atresia in porcine granulosa cells are pursued. The aim of this study was to decipher the roles of BimEL phosphorylation during porcine follicular atresia.

## 2. Materials and Methods

### 2.1. Classification of Healthy, Slightly Atretic, and Atretic Follicles and Recovery of Granulosa Cells

The ovaries from gilts aged about 5 months old were collected at a local abattoir and transported to the laboratory in a vacuum flask (30–35 °C) containing sterile physiological saline within 2 h. Ovaries were washed twice with sterile physiological saline (37 °C) containing 100 IU/L penicillin and 50 mg/L streptomycin. Healthy, slightly atretic, and atretic follicles were classified according to previously established morphological criteria [7,8,23,24]. Briefly, healthy follicles were defined as vascularized theca internal and clear amber follicular fluid with no debris. The follicles lacking any of these criteria were classified as atretic. The slightly atretic and atretic follicles had gray theca internal and flocculent follicular fluid in varying degrees. Follicular contents were punctured by hypodermic needle, and cumulus–oocyte complex and ovarian tissue were discarded under a stereo microscope. Granulosa cells were harvested by centrifuging.

### 2.2. Granulosa Cell Culture and Experimental Design

Porcine granulosa cells were cultured as previously described [8,13,23]. Briefly, the granulosa cells from healthy follicles (2–5 mm in diameter) were isolated by puncturing follicles with a 25-gauge hypodermic needle. The granulosa cell masses were recovered by pipette under stereoscope and cultured in DMEM/F12 supplemented with 100 IU/L penicillin and 50 mg/L streptomycin after washing thrice for different treatments. The cells that were suspended during culture in this media were defined as primary granulosa cells. To obtain adherent monolayer granulosa cells, the cells were gently washed thrice and then cultured in DMEM/F12 supplemented with 10% fetal bovine serum (FBS), 100 IU/L penicillin, and 50 mg/L streptomycin at 37 °C in humidified air with 5% CO_2_ for 24 h. The cells were passaged upon reaching confluence. Granulosa cells from about 10 ovaries can be selected and cultured in 10 dishes with a diameter of 35 mm as primary granulosa cells, and the same amounts of cells cultured in 4 dishes with a diameter of 100 mm as adherent granulosa cells.

**Figure 1:**
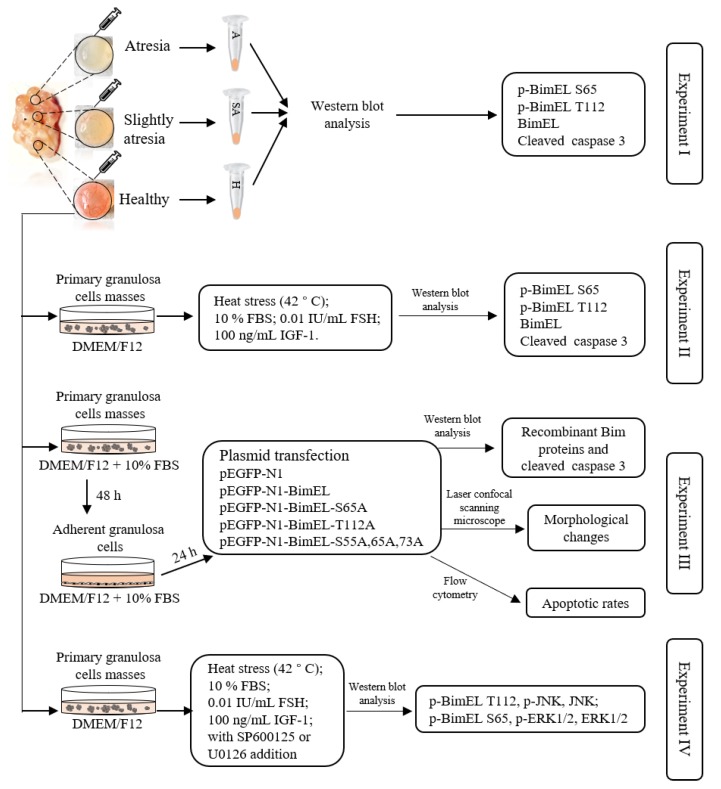
The workflow schematic of this study. Experiment I: Both levels of p-BimEL S65 and p-BimEL T112 in the granulosa cells from healthy, slightly-atretic, and atretic follicles were compared. Experiment II: The effects of proapoptotic factor (heat stress) and prosurvival factors (serum, FSH, and IGF-1) were observed on the levels of p-BimEL S65 and p-BimEL T112. Experiment III: The effects of phosphorylation at Ser65 and Thr112 were observed during apoptosis in porcine granulosa cells. Experiment IV: The potential pathways were suggested to induce the levels of p-BimEL S65 and p-BimEL T112 by the prosurvival or proapoptotic factors. Primary granulosa cells masses = granulosa cells recovered from follicles; adherent granulosa cells = monolayer adherent granulosa cells after in vitro culture.

Figure 1 outlines the whole design of this experiment. Experiment I: Both levels of p-BimEL S65 and p-BimEL T112 in the granulosa cells from healthy, slightly-atretic, and atretic follicles were compared. Experiment II: The effects of proapoptotic factor (heat stress) and prosurvival factors (serum, FSH, and IGF-1) were observed on the levels of p-BimEL S65 and p-BimEL T112. Primary granulosa cell masses were treated with 10% FBS, FSH (0.01 IU/mL), or IGF-1 (100 ng/mL) for 24 h. Heat stress was carried out according to the description in a previous study [25]. Briefly, the primary granulosa cells masses were incubated at 42 °C in humidified air with 5% CO_2_ for 12 h. Both levels of p-BimEL S65 and p-BimEL T112 were compared among different treatments. Experiment III: The effects of phosphorylation at Ser65 and Thr112 were observed during apoptosis in porcine granulosa cells. BimEL and BimEL mutation vectors were constructed and transfected into porcine granulosa cells. The levels of recombinant Bim proteins and morphological changes of granulosa cells were compared among the cells transfected by different vectors. The apoptotic rates were examined by flow cytometry. Experiment IV: The potential pathways were suggested to induce the levels of p-BimEL S65 and p-BimEL T112 by the prosurvival or proapoptotic factors.

### 2.3. Construction of BIM DNA Mutant Vectors and Transfection

The PCR-amplified full-length porcine BimEL cDNA was constructed into pEGFP-N1 (Clontech). The reading frame of BimEL cDNA was connected with enhanced GFP to obtain a recombinant pEGFP-N1-BimEL plasmid. The plasmid was propagated using Trans1-T1 (Transgen Biotech, Beijing, China) as the host strain and purified with a Plasmid MiniPrep Kit (EM111-01, Transgen Biotech), followed by sequencing to confirm the open reading frame. Mutations of BimEL phosphorylation sites were performed by a Fast Mutagenesis System (FM111, Transgen Biotech), and primers for mutations were designed according to the manufacturer’s instructions. Primers used in this experiment are shown in Table 1. Based on the pEGFP-N1-BimEL plasmid, porcine BimEL Ser 65 was mutated to alanine (BimEL-S65A), Thr 112 to alanine (BimEL-T112A), and Ser 55/65/73 to alanine (BimEL-S55A65A73A).

NucleofectorTM 2b Device (Lonza, AAB-1001, Cologne, Germany) and Basic Nucleofector^TM^ Kit for Primary Mammalian Fibroblasts (Lonza, VPI-1002, Cologne, Germany) were used for plasmid transfection. The transfection protocol followed the manufacturer’s instruction. Briefly, monolayer adherent granulosa cells were harvested after reaching confluence and divided into 6 equal parts (about 2 × 10^6^ cells each) named as CN (with no plasmid but treated the same with other groups), vector (pEGFP-N1 transfection), BimEL (pEGFP-N1-BimEL transfection), BimEL-S65A (pEGFP-N1-BimEL-S65A transfection), BimEL-T112A (pEGFP-N1-BimEL-T112A transfection), and BimEL-S55A65A73A (pEGFP-N1-BimEL-S55A65A73A transfection). A total of 100 µL Nucleofector™ solution and 1.5 µg plasmid DNA were added to each group except for the CN; then, the cuvette with cell/DNA suspension was inserted into the Nucleofector™ Cuvette Holder to carry out the Nucleofector™ Program V-024 individually. After that, a total of 600 µL of DMEM media were added into each group, and the samples were transferred immediately into six-well plate (final volume 2.0 mL media per well). Finally, the cells were cultured for 24 h or 48 h in humid air with 5% CO2 at 37 °C for further analysis.

### 2.4. Observation of Cell Morphologies in Porcine Granulosa Cells after Transfection with Different BIM Mutant Vectors

The granulosa cells were first transfected with BimEL, BimEL-S65A, BimEL-T112A, and BimEL-S55A65A73A vectors and cultured in cell slides with or without QVD-OPH (a general caspase inhibitor, HY-12305, MCE, Shanghai, China) treatments, respectively. After that, the cells were fixed with 4% paraformaldehyde, and the nuclei were stained with Hoechst 33342. The samples were analyzed for morphological changes in cells and nuclei by a laser confocal scanning microscope (Nikon C1 standard detector, Nikon, Japan).

### 2.5. Protein Extraction and Immunoblotting

The cells were harvested and washed once in PBS, then lysed on ice for 30 min with RIPA buffer (CST, 9806) and supplemented with 1% (*v*/*v*) protease inhibitor Cocktail (HY-K0010) and 1% (*v*/*v*) phosphatase inhibitors (Cocktail I, HY-K0021; Cocktail II, HY-K0022; and Cocktail III, HY-K0023) purchased from MCE (MedChemExpress, Shanghai, China). Western blotting was performed as described previously [13]. Protein concentrations were determined by BCA protein assay kit (Transgen Biotech), and equal amounts of proteins (15-50 μg/lane) were separated by SDS-PAGE (12% acrylamide running gel) and transferred to a nitrocellulose membrane (BioTrace™ NT, Pall Corp, FL, USA). The following antibody was used in this experiment: phospho-Bim (Thr56, Thr116) (PA5-64655, Thermo Fisher Scientific Shanghai, China); phospho-Bim (Ser69) (4581), p-JNK (9251S), p-ERK1/2 (9101S), cleaved caspase 3 (9664S), and Bim (C34C5) were all purchased from Cell Signaling Technology (Shanghai, China); ERK1/2 (abs130092), JNK1/2 (abs131832), and Actin (abs132001) were purchased from Absin (Shanghai, China). The Western blotting images were processed using Image J software (National Institutes of Health, Bethesda, MD, USA). The antibodies were diluted at the recommended ratio with Beyotime (P0256, Shanghai, China) diluent.

### 2.6. Histology

Porcine ovaries (2–5 mm in diameter) were fixed overnight in 4% phosphate-buffered formaldehyde at 4 °C for 7 days and then embedded in paraffin. Randomly selected sections (5 μm each) were used for subsequent Hematoxylin–eosin, TUNEL, and immunohistochemical analyses. Hematoxylin and eosin were purchased from Solarbio (Beijing, China).

Apoptosis was analyzed by TUNEL (In Situ Cell Death Detection Kit, TMR red, Roche, Shanghai, China) according to the manufacturer’s protocol. Briefly, after deparaffinization and rehydration, sections were incubated with proteinase-K (20 g/mL) for 15 min at room temperature and quenched in 3% H_2_O_2_ in PBS for 10 min (to block endogenous peroxidase). They were then incubated in a humidified chamber with equilibration buffer for 5 min and finally incubated with terminal deoxynucleotidyl transferase for 1 h at 37 °C. Negative control slides were incubated as described above, without the final addition of terminal deoxynucleotidyl transferase. After TUNEL reactions were complete, slides were washed in PBS, sealed under coverslips with nail varnish and examined under fluorescence microscopy.

Immunohistochemical analysis was performed as previously described [23]. Briefly, phospho-Bim (Ser69), 1:200, was used to detect porcine phospho-Bim-Ser65 expression. HRP-conjugated anti-rabbit antibody (1:100, Zhongshan Biotechnology, Beijing, China) was applied for 1 h at room temperature. The binding of primary antibody was visualized using diaminobenzidine for 3–5 min. The phospho-Bim-Ser65 labeling was examined using a Leica microscope, and images were recorded (Leica DC 200 digital camera; Leica, Wetzlar, Germany).

### 2.7. Apoptosis Assay by Fluorescence-Activated Cell Sorter

QVD-OPH (20 μM) was added immediately after transfection. After 24 h, QVD-OPH was withdrawn by changing the culture media, and another 24 h later, the cells were harvested and stained with PE-conjugated Annexin V and 7-AAD in a binding buffer for 15 min as described previously [26,27]. GFP-positive cells were gated for apoptosis analysis by a fluorescence-activated cell sorter (FACS, Becton Dickinson, Franklin Lakes, NJ, USA), and the data were analyzed by FlowJo_V10 software.

### 2.8. Statistical Analyses

All data are presented as the mean ± SD and were analyzed using a one-way ANOVA with SPSS 22 (IBM, SPSS, Chicago, IL, USA) for Windows. The ANOVA followed by a post hoc Dunnett’s test was used to determine significant differences between different groups. Differences were considered statistically significant at *p* < 0.05.

## 3. Results

### 3.1. Subsection

#### 3.1.1. The levels of P-BimEL-S65 were Decreased while P-BimEL-T112 were Increased in Granulosa Cells during Follicular Atresia

**Figure 2:**
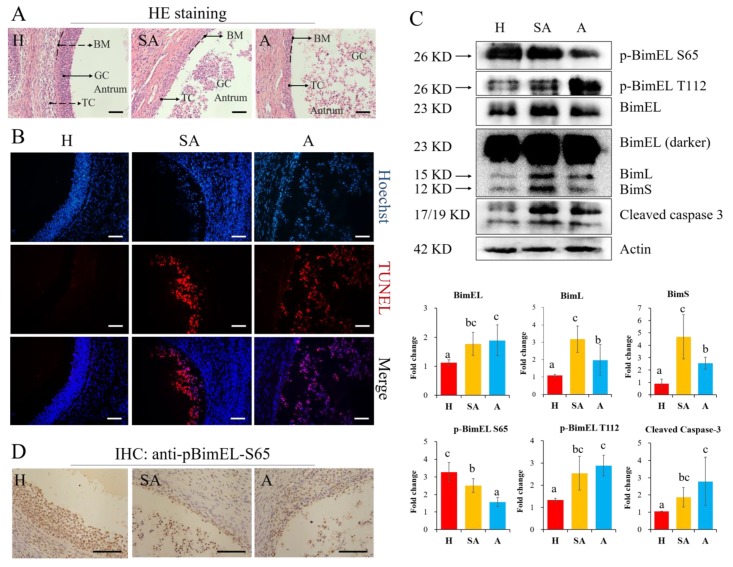
Levels of p-BimEL-S65 were decreased while p-BimEL-T112 were increased in granulosa cells during follicular atresia. (**A**) Hematoxylin–eosin (HE) staining of porcine healthy, slightly atretic and atretic follicle. Scale bar = 100 μm. BM = basement membrane, GC = granulosa cell, TC = theca cell. (**B**) Hoechst and TUNEL staining of follicles with different status. Scale bar = 100 μm. (**C**) Granulosa cells obtained from porcine healthy, slightly atretic, and atretic follicles were analyzed by Western blotting. Actin is the loading control. Column charts show the quantitative analysis of p-BimEL S65, p-BimEL T112, BimEL, BimL, BimS, and cleaved caspase-3. The data are representative of three independent experiments. Values are expressed as the means ± SD of three separate experiments. The bars are labeled with completely different letters indicating significant difference, *p* < 0.05. (**D**) p-BimEL-S65 protein was localized with specific antibodies by immunohistochemistry (IHC). Scale bar = 100 μm. H = healthy follicle; SA = slightly-atretic follicle; A = atretic follicle, p-BimEL S65 = phosphor-BimEL-Ser65; p-BimEL T112 = phosphor-BimEL-Thr112.

To confirm the follicular status identified by the morphology criteria, HE staining was performed in healthy, slightly-atretic, and atretic follicles. The results showed that all granulosa cells tightly lined the basement membrane in healthy follicles, but cells began to diffuse away from the basement membrane in slightly atretic follicles. More seriously, the cells drifted to the follicular antrum in the atretic follicles (Figure 2A). Furthermore, granulosa cells in healthy follicles rarely showed TUNEL signal, while slightly atretic and atretic follicles exhibited strong apoptotic signals (Figure 2B). The changes of p-BimEL S65, p-BimEL T112, and other related proteins in granulosa cells from porcine healthy, slightly atretic, and atretic follicles were analyzed by Western blotting. The results showed that the level of p-BimEL S65 was higher, while the level of p-BimEL T112 was lower in healthy follicles than in atretic ones. Conversely, p-BimEL S65 was lower, while the level of p-BimEL T112 was more highly expressed in atretic follicles compared with the healthy ones (Figure 2C). In addition, the levels of three Bim isoforms (BimEL, BimL, and BimS) and cleaved caspase-3 were all higher in slightly atretic and atretic follicles than those in healthy follicles. Immunohistochemical results of porcine ovarian tissue further confirmed that the expression profile of p-BimEL-S65 was similar to the above results (Figure 2D).

#### 3.1.2. Effects of Proapoptotic and Survival Factors on the Levels of P-BimEL S65 and P-BimEL T112 in Cultured Granulosa Cells

**Figure 3:**
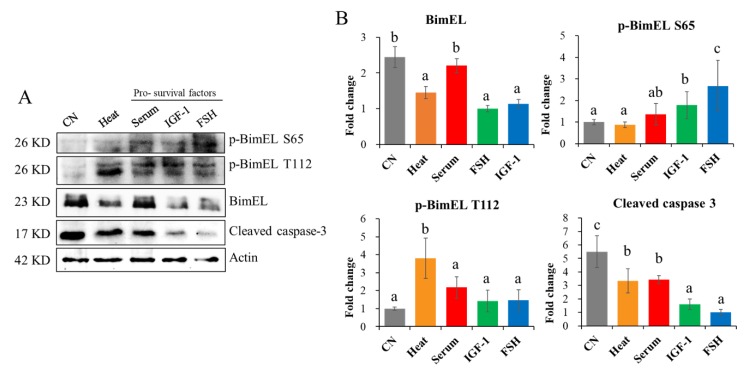
Effects of proapoptosis and prosurvival factors on the levels of p-BimEL S65, p-BimEL T112, and other related proteins in cultured granulosa cells.(**A**) Protein levels of porcine primary granulosa cells treated with heat stress, serum, IGF-1 or FSH. CN = cultured without treatment; Heat = heat stress treatment at 42 °C for 12 h; serum = 10% fetal bovine serum treatment for 24 h; IGF-1 = treated with 100 ng/mL IGF-1 for 24 h; FSH = treated with 0.01 IU/mL FSH for 24 h. (**B**) Quantitative analysis of BimEL, p-BimEL S65, p-BimEL T112, and cleaved caspase-3. p-BimEL S65 = phosphor-BimEL-Ser65; p-BimEL T112 = phosphor-BimEL-Thr112. Values are expressed as the means ± SD of three separate experiments. The bars labeled with completely different letters indicate significant difference, *p* < 0.05.

Proapoptotic factors like heat stress in summer may induce granulosa cell apoptosis and cause follicular atresia, while prosurvival factors like FSH and IGF-1 can promote granulosa cell survival and proliferation. Therefore, the effects of these factors on the levels of p-BimEL S65 and p-BimEL T112 in granulosa cells were investigated. Porcine primary granulosa cells were cultured in vitro and treated with proapoptotic (heat stress) or survival factors (serum supply, IGF-1, and FSH), and the levels of p-BimEL S65, p-BimEL T112, and other related proteins were detected (Figure 3A). The results showed that the level of BimEL in granulosa cells was decreased after heat stress, FSH, and IGF-1 treatment compared with the CN and serum treatment groups. The levels of p-BimEL S65 were elevated after IGF-1 and FSH treatments compared with the CN and heat stress (Figure 3B). The level of p-BimEL T112 was raised after 12 h of heat stress at 42 °C (Figure 3B) compared with the CN and the prosurvival factor treatment groups. The level of cleaved caspase-3 was decreased after the serum, IGF-1, and FSH treatments compared with the CN (Figure 3B).

#### 3.1.3. Effects of BimEL Phosphorylation Site Mutation on the Apoptosis of Porcine Granulosa Cells

**Figure 4:**
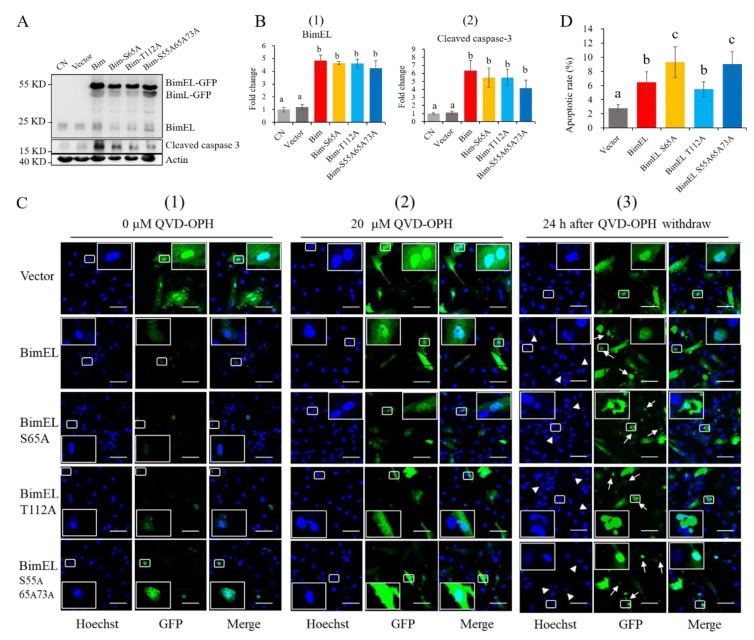
Effects of BimEL phosphorylation sites mutation on apoptosis of porcine granulosa cells. Porcine granulosa cells were transfected with vector (pEGFP-N1), BimEL (pEGFP-N1-BimEL), BimEL-S65A (pEGFP-N1-BimEL-S65A), BimEL-T112A (pEGFP-N1-BimEL-T112A), and BimEL-S55^A^65^A^73^A^ (pEGFP-N1-BimEL-S55^A^65^A^73^A^) plasmids, and the CN was the transfection control. Values are expressed as means ± SD from five independent experiments. The bars labeled with completely different letters indicate significant difference, *p* < 0.05. (**A**) The levels of recombinant Bim-GFP proteins were compared among the porcine granulosa cells transfected with different plasmids. The cells were harvested for Western blot after transfection for 24 h. (**B**) The results of quantitative analysis of BimEL (B-1) and cleaved caspase-3 (B-2) levels in different groups. (**C**) The treatment of QVD-OPH increased the numbers of green positive cells after transfection with BimEL and BimEL phosphorylation site mutations. The cells from each plasmid transfection were equally divided into three groups and were cultured in one slide. (C-1) The cells in the 0 μM QVD-OPH (DMSO control). (C-2) The cells of 20 μM QVD-OPH groups were cultured for 24 h. The Cells (C-3) were cultured in the medium containing 20 μM QVD-OPH for 24 h and then cultured in the same medium without QVD-OPH for another 24 h. After culturing, the cells were fixed with 4% paraformaldehyde and stained with Hoechst 33342. The samples were analyzed with a laser confocal scanning microscope. Representative images are shown. Scale bar = 100 μm. Triangle indicates the fragmented or shrunk nuclei. Arrows point out the shrinkage cells. (**D**) Apoptotic rates of green positive cells after transfection with BimEL and BimEL with different phosphorylation site mutations. QVD-OPH (20 μM) was administrated immediately after transfection. After 24 h, QVD-OPH was taken out, and the cells were harvested after an additional 24 h for Annexin V and 7-AAD staining, followed by flow cytometry analysis. The green positive cells were further analyzed for Annexin V and 7-AAD. The percentages of apoptotic cells (Annexin V positive) from each group were compared.

To further study effects of the two key sites of phosphorylation in BimEL on granulosa cells, BimEL and BimEL phosphorylation site mutation plasmids were constructed. The plasmids of wild-type BimEL, BimEL-S65A, BimEL-T112A, or BimEL-S55^A^65^A^73^A^ were transfected into porcine granulosa cells with about 30% efficiency. The results demonstrated that BimEL-GFP and BimL-GFP in the four transfections were all expressed, while endogenous Bim expression levels did not change compared with the pEGFP-N1 vector and transfection control (CN) (Figure 4A and B-1). Notably, levels of cleaved caspase 3 were elevated in the four groups (Figure 4B-2).

After 24 h of transfection, the green positive cells were seldom found in the groups transfected with BimEL, BimEL-S65A, BimEL-T112A, and BimEL-S55^A^65^A^73^A^ plasmids compared to those in the pEGFP-N1 vector. Moreover, the positive cells were almost wrinkled with fragmented or shrunk nuclei (Figure 4C-1). However, the amount of positive green cells increased in BimEL and its mutant groups when the cells were treated with 20 μM QVD-OPH (inhibitor of apoptosis) for 24 h immediately after transfection, and most green cells displayed normal morphologies (Figure 4C-2). After 24 h of QVD-OPH withdrawal, most green cells in BimEL and its mutant groups exhibited apoptosis, including cellular shrinkage, plasma membrane blebbing, and cellular or nuclear fragmentation (Figure 4C-3).

Apoptotic rates of the cells transfected with BimEL, BimEL-S65A, BimEL-T112A, or BimEL-S55^A^65^A^73^A^ plasmids were compared by flow cytometry assay. The results demonstrated that apoptotic rates of cells in BimEL and its mutant groups were all higher than that of the pEGFP-N1 vector. Furthermore, BimEL-S65A and BimEL-S55^A^65^A^73^A^ groups had higher apoptotic rates than others, while BimEL-T112A had no difference with the BimEL group in apoptosis (Figure 4D).

#### 3.1.4. Regulations of P-BimEL S65 and P-BimEL T112 in Porcine Granulosa Cells

**Figure 5:**
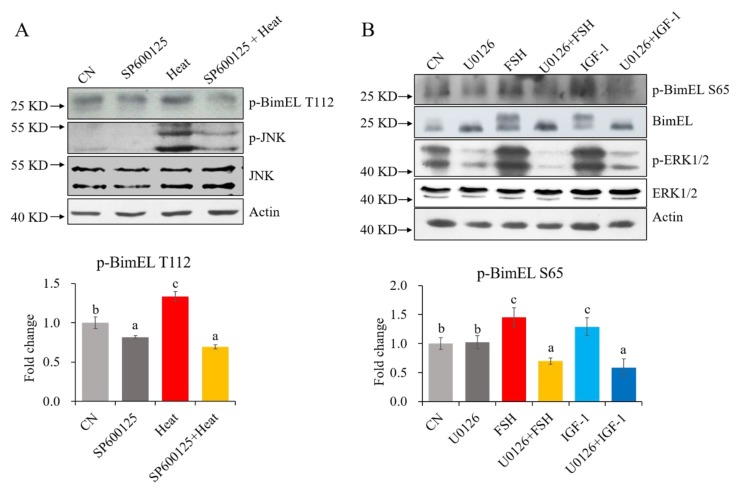
The possible pathways of p-BimEL S65 and p-BimEL T112 regulated by different factors in porcine granulosa cells. (**A**) The treatment of SP600125 blocked the increase of BimEL T112 phosphorylation induced by heat stress. (**B**) The treatment of U0126 hindered the increase of BimEL S65 phosphorylation induced by FSH or IGF-1. CN = untreated, Heat = heat stress at 42 °C for 12 h, IGF-1 = treated with 100 ng/mL IGF-1 for 24 h, FSH = treated with 0.01 IU/mL FSH for 24 h, U0126 = treated with 20 μM U0126 for 24 h, SP600125 = treated with 20 μM SP600125 for 12 h; p-BimEL S65 = phosphor-BimEL-Ser65; p-BimEL T112 = phosphor-BimEL-Thr112. The cells were harvested, and equal amounts of proteins were used for immunoblotting. Values are expressed as the means ± SD of three separate experiments. The bars labeled with completely different letters indicate significant difference, *p* < 0.05.

Previous studies showed that BimEL could be phosphorylated at Ser65 by ERK1/2 [28] and at Thr112 by JNK [16]. To confirm whether p-BimEL S65 and p-BimEL T112 are regulated by ERK1/2 and JNK in granulosa cells, the porcine primary granulosa cells were treated by heat stress, FSH, and IGF-1 together with different specific inhibitors of cellular pathways. The present results show that heat stress increased both the levels of p-JNK and p-BimEL T112, but SP600125 prevented their changes. Similarly, FSH and IGF-1 treatments increased p-ERK1/2, p-BimEL S65, and total BimEL phosphorylation levels; however, U0126 could block these shifts (Figure 5). The results indicate that BimEL S65 and BimEL T112 can be phosphorylated by the ERK1/2 and JNK pathway, respectively, in porcine follicular granulosa cells.

## 4. Discussion

Our results illustrate the dynamic changes of BimEL phosphorylation during porcine follicular atresia, and its biofunctions vary with phosphorylation sites regulated by different pathways. The survival factors induce phosphorylation at S65 and inhibit granulosa cell apoptosis through the ERK pathway, whereas the proapoptotic factor stimulates phosphorylation at T112, leading to granulosa cell apoptosis by the JNK pathway.

It is well-known that apoptosis of granulosa cells contributes to ovarian follicular atresia [29], and our previous research confirmed that the level of BimEL was higher in porcine atretic follicles than in healthy ones [23]. This study shows that the apoptotic granulosa cells detach from the basement membrane and enter the follicular antrum (Figure 2A,B). However, post-translational modification of BimEL in follicles is poorly understood. According to our current knowledge, this research is the first to examine the changes in BimEL phosphorylation sites during mammalian follicular atresia. Phosphorylation levels of BimEL at S65 and T112 are inversed in granulosa cells from healthy and atretic follicles. Immunohistochemical results of porcine ovarian tissue further confirmed the expression profile of p-BimEL-S65; however, anti-pBimEL-T112 antibody is not suitable for immunohistochemistry. A previous study on K562 cells reported that phosphorylation at S65 promoted degradation of BimEL to inhibit apoptosis [14], and BimEL degradation requires the ERK-dependent pathway [14,15]. Our recent research demonstrated that melatonin promotes ubiquitination of phosphorylated BimEL in porcine granulosa cells [13]. Nevertheless, phosphorylation at T112 promoted separation of BimEL from DCL8 to induce apoptosis [26]. Alternative splicing can delete sequences derived from exon 3 or exons 3 and 4 to create BimL and BimS, respectively. We showed that the two kinds of Bim isoform, BimL and BimS, exhibited the same pattern as BimEL in granulosa cells during follicular atresia. Although the relative expression levels of BimL and BimS were lower than BimEL, they too may also have strong apoptotic activity [30]. This suggests that these two splices may also be involved in the apoptosis of granulosa cells. Bim-targeting cancer therapies may provide more effective and unique tumor management modalities in the future [31]. For example, miR-25 can regulate apoptosis by targeting Bim in human ovarian cancer and may be a potential therapeutic target for ovarian cancer [32].

BimEL was first demonstrated to be a phospho-protein in IL-3-stimulated BaF3 cells [33]. BimEL in ovarian granulosa or other cells could be phosphorylated by proapoptotic factors like UV and heat stress [26] and also by survival factors such as IGF-1 [28,34], FSH [13,23], and melatonin [13]. Mammalian fertility is reduced during heat exposure in the summer because heat stress caused by high environmental temperature impairs folliculogenesis, leading to ovulation of low-quality oocytes [35]. Heat stress was also reported to alter follicular dynamics [36], steroidogenic ability [37], granulosa cell function [38], and oocyte maturation [39,40], which contributes to low reproductive efficiency in mammals during the summer [25]. Withdrawing serum was reported to induce neuronal cell and R28 cell apoptosis by increasing caspase-3/7 activity and promoting cleavage of caspase-9 and -3 [28,41]. Nevertheless, Bim^KO^ mouse embryonic fibroblasts are resistant to serum withdrawal-induced apoptosis [12]. However, limited information is available on the dynamics, functions, and regulations of its phosphorylation sites in granulosa cells during follicular atresia. In cultured granulosa cells, we found that heat stress could increase phosphorylation of BimEL at T112, while serum, FSH, or IGF-1 treatment promoted phosphorylation of BimEL at S65. Previous studies revealed that survival factors can phosphorylate BimEL at S65 through the ERK pathway [14,18]. In addition, we noticed that serum treated granulosa cells showed a higher level of cleaved caspase 3 than that in the FSH or IGF-1 groups, which indicated that serum may be not an eminent prosurvival factor.

In the present results, overexpression of BimEL or BimEL with different phosphorylation site mutations increased granulosa cells apoptosis in vitro, which is consistent with our previous results [23]. The levels of apoptosis were similar when the cells were transfected with BimEL or BimEL-T112A. These results agree with research on the 293T cell [26]. However, the apoptotic rate was increased when the serine at the 65th amino acid residue in BimEL was replaced by alanine to prevent its phosphorylation. This was consistent with reports that BimEL without phosphorylation of S55A, S65A, and S100A had strong interactions with BAX to enhance its proapoptotic activity [42]. In this experiment, BimEL-S55^A^65^A^73^A^ increased the apoptotic rate of porcine granulosa cells, which was consistent with the previous results from BimEL S55/65/73A mutant mice [11]. In mice, the mutation of T112 in the *Bim* gene decreased binding of Bim to the antiapoptotic protein Bcl2, increasing cell survival because the binding of Bim to Bcl2 contributes to Bim-induced apoptosis [43]. In contrast, mutation of the phosphorylation sites S55, S65, and S73 increased apoptosis because of reduced proteasomal degradation of Bim [11]. Although the three phosphorylation sites are implicated in the regulation of Bim stability, the S65 phosphorylation site may play a vital role in this process. Activated ERK1/2 binds to human BimEL via the DEF domain and phosphorylates Ser69 (same as pig BimEL Ser65), which in turn induces a conformational change allowing phosphorylation at a second site [18]. Phosphorylation at S69 can also promote phosphorylation at additional sites on Ser 93/94/98 by ribosomal S6 kinase (Rsk1/2) within a conserved phosphodegron motif recognized by the F-box protein beta-transducin repeat containing E3 ubiquitin-protein ligase (βTrCP) [44].

BimEL can be phosphorylated by several MAP kinases (including ERK, JNK, and p38 isoforms) to regulate its activity [11]. Previous research reported that the ERK1/2-dependent phosphorylation of BimEL promotes its rapid dissociation from Mcl-1 and Bcl-xL in CR1-11 cells. The dissociation of BimEL from Mcl-1 requires phosphorylation of BimEL at S65 through the ERK1/2 pathway [12]. IGF-1 was reported to induce BimEL phosphorylation through ERK1/2 in an R28 rat retinal neuronal precursor cell line [28]. Gliotoxin and the supernatant of *Aspergillus fumigatus* activated the JNK pathway to induce BimEL phosphorylation at S100, T112, and S114, causing apoptosis in mouse fibroblasts, alveolar epithelial cells, and human bronchioles [16]. Exposure to environmental stress, including UV radiation, caused JNK-dependent phosphorylation of BimEL in 293T cells [26]. Herein, we confirmed that p-BimEL S65 and p-BimEL T112 in follicular granulosa cells were also regulated by ERK1/2 and JNK, respectively.

**Figure 6:**
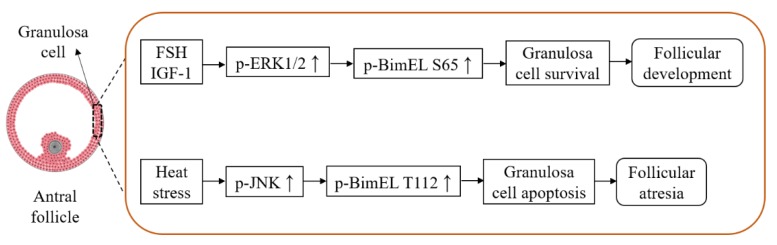
Hypothetical model of functions and regulations of p-BimEL S65 and p-BimEL T112 during follicular atresia.

Based on our results, a hypothetical model of BimEL phosphorylation participating in animal follicular atresia is presented in Figure 6. In the antral follicles, survival factors like FSH and IGF-1 promote phosphorylation of BimEL S65 via the ERK pathway inhibiting granulosa cell apoptosis, and, therefore, could prevent follicular atresia. Nevertheless, harmful factors like heat stress or insufficient growth factors will stimulate the phosphorylation of BimEL T112 via the JNK pathway inducing granulosa cell apoptosis, and contribute to follicular atresia. The present results increased our knowledge about the role of BimEL phosphorylation during follicular atresia. Moreover, it may be helpful in improving efficiency of assisted reproductive technologies and treatment of infertility caused by ovarian dysplasia.

## Figures and Tables

**Table 1:** Primers used to amplify pig BimEL and BimEL mutation.

Name	Primer Sequence	
BimEL	Forward	gaattcatggcaaagcaaccttccgatg
	Reverse	ggatccgcaatgtaagggggagggagggt
BimEL-S65A	Forward	ccactggccccaccgacc*GCC*cctggcccctttgctacc
	Reverse	*GGC*ggtcggtggggccagtgggccctgggggctgccttg
BimEL-T112A	Forward	aaatcaacacaa*GCC*ccaagtcctccttgccaagcc
	Reverse	acttgg*GGC*ttgtgttgatttgtcacaactcatggg
BimEL-S55^A^65^A^73^A^	S55A-Forward	gaccgctgcccccaaggc*GCC*ccccagggcccactg
	S55A-Reverse	*GGC*gccttgggggcagcggtccccttctccttccgg
	S73A-Forward	tggcccctttgctaccaga*GCC*ccgcttttcatcttcgtg
	S73A-Reverse	*GGC*tctggtagcaaaggggccaggggcggtcggtgg

Mutation of BimEL-S65A and BimEL-T112A were based on pEGFP-N1-BimEL, mutation of BimEL-S55^A^65^A^73^A^ (S55A, S65A, and S73A) was based on pEGFP-N1-BimEL-S65A.
